# Achieving dedicated programmes and funding for eye care

**DOI:** 10.2471/BLT.18.031018

**Published:** 2018-10-01

## Abstract

Eye care is increasingly being incorporated into national health strategies in many countries. Haroon Awan talks to Fiona Fleck.

Q: How did you become interested in eye care?

A: I had a lot of inspiration from my family. I grew up in Kenya where my father was the chief ophthalmologist at the health ministry. He would often take me to the eye clinic during the school holidays and I got to know the nurses and ophthalmic clinical officers in the eye clinic. They would tell me a lot about their work, and so I developed an interest in eye care. It was a close community and I came across many people from different indigenous and ethnic backgrounds whom my father had treated and whenever we met them, they would say how grateful they were to have had such a good eye doctor.

Q: What was the eye-care situation like in Kenya at the time?

A: Kenya had few ophthalmologists in the country. The main burden of work was done by ophthalmic clinical officers working in urban districts and rural areas. They were trained in cataract and minor eye surgery and they could refer patients to the central hospital. I completed the ophthalmology residency training programme at that hospital. There, I was exposed to many of the issues confronting eye care in Kenya at the time, such as the challenges of ophthalmic planning, the supply chain for glasses, estimating the needs for a particular region, as well as, the needs for clinical and surgical services. As residents, we spent time with two or three provincial coordinators where, in one region trachoma might be more prevalent, while in others cataracts was more common. One of the main challenges was that we did not have enough ophthalmologists to cover many of the provincial and district hospitals. 

Q: How did you become involved in the development of Pakistan’s National Strategy for Eye Care? 

A: I moved from to Pakistan from Kenya in 1990, after my post-graduate training and joined Al-Shifa Eye Hospital, in Rawalpindi. There I was nominated to join the National Committee for the Prevention of Blindness, tasked by the health ministry to develop strategies for eye health promotion and prevention, and for the control of avoidable blindness. I realized that while the clinical aspects of eye care came naturally to senior clinical professionals, planning for eye-care programmes at the provincial and national level beyond hospital services was new for them. Planning for eye-care programmes was something I had learnt and practiced in Kenya, so I offered my suggestions.

Q: What was the response?

A: The response was positive. In 1993, I joined the team led by the national coordinator for eye care, Professor Mohammad Daud Khan, working with a Ministry of Health team of provincial coordinators to draft the first 5-year plan for Pakistan (1994–1998) for the prevention of blindness. The first national blindness survey conducted between 1989 and 1990, with WHO support, found a high prevalence of blindness at 1.78%. There were about 500 ophthalmologists in a population of about 130 million people, about 50% of district hospitals had some form of eye-care services and about 140 000 cataract surgeries were performed each year. Moreover, there was no primary eye care; there were no optometrists; and very few ophthalmic technicians (ophthalmic assistants). That was our starting point.

Q: Much eye care in low-income countries is provided by charities and nongovernmental organizations (NGOs) that are not necessarily working with the government or the health ministry. Why is that?

A: This is partly because of inadequate eye care services in the public sector and partly by the way eye-care services were funded in the past. In the 1990s, when I came to Pakistan, eye-care services were fragmented. Most government and private eye care services at the time were concentrated in urban centres, while very few government district hospitals in the rural areas had any eye care services. NGOs and faith-based organizations supplemented government eye care services and often went to hard-to-reach areas to provide free services to those most in need, especially where there were no government facilities for eye care. Their service delivery approach was linked to their respective organizational strategies. That meant that NGOs working in eye care developed resource mobilization strategies mainly based on the idea of, ‘Give us so many dollars and we will do so many cataract surgeries’. This appealed to donors, because they could give x dollars and cataract blindness could be eliminated in x number of people. So while the government in Pakistan was more interested in developing infrastructure for static facilities, NGOs were making vertical interventions in a specific area.

Q: Are governments and NGOs working more closely together now?

A: Yes. National committees for eye health in many development countries now have a broader scope and include participation from NGOs and faith-based organizations to support the committees and provide direction to eye-care programmes. It is a mutually reinforcing relationship, in which the government benefits from the support of the NGOs and vice versa. For example, Al-Shifa Trust Eye Hospital in Pakistan, has four major hospitals across the country, one in each province, offering high quality specialist care. In addition, the Layton Rahmatullah Benevolent Trust has over 20 eye hospitals providing quality eye-care services free of charge and is the largest safety net for poor people who need eye care in Pakistan. These NGO strategies have been developed in line with national guidelines and in collaboration with the National Committee for Eye Health. Over time, and with the launch of the VISION 2020: the Right to Sight initiative in 1999 and the subsequent WHO health systems framework and *Universal eye health: a global action plan 2014–2019*, national committees have started to take a health systems approach to eye-care programmes.

Q: Can you tell us about your work building capacity for eye care in other countries?

A: WHO has been urging countries in WHO’s Eastern Mediterranean region to develop their own national eye health programmes, but there are many challenges. When the *global action plan 2014–2019* was launched, many countries wanted to develop their own eye health plans. I worked with colleagues in the health ministries in Bangladesh, Jordan, Kuwait, Libya, Oman, Qatar and Saudi Arabia. We developed 5-year eye health plans that link eye care to the government’s health strategies. 

Q: What challenges did you face? 

A: National counterparts for eye care were not really aware of their own country’s health sector strategies, as the national eye health plans were being developing in isolation. Eye-care programmes were not engaging enough with their respective health ministries. Human resources for health plans did not include ophthalmologists, who were lumped under ‘other health professionals’. In addition, while many countries had fairly good health information and some improved eye-care indicators, eye-care programmes were also not engaging with the health information system. Eye health would be classed as ‘other conditions’ in reports generated for policy-makers, so there would be no specific statistics on eye health. As a result, no resources were allocated specifically for eye care. Finally, in almost all the countries where I worked, the national committees for eye health were not familiar with the health ministries’ planning and budgeting process, including how resources were allocated to different programmes, thus missing out on precious funding for eye care.

Q: How did you and your colleagues overcome the challenges?

A: In Pakistan, for example, we discussed the situation with the health ministry and pursued financing for eye health through the government’s processes for planning and budgeting for new projects. As a result, the National Eye Health Plan 2005–2010 was allocated US$ 50 million from the government of Pakistan. It was the first time that eye care was allocated dedicated financing and this opened the door for regular eye health financing. Now each province allocates dedicated funding to health eye. Since then, also, national and regional eye health leaders have been mobilizing resources for comprehensive eye care through the government’s routine planning and budgeting.

Q: Why has eye care been treated separately from other health care? 

A: In some countries where trachoma is endemic, such as in the African region, trachoma is often the national or regional health priority for eye care. Similarly, in some countries in the Eastern Mediterranean region, the focus of eye care has been on cataract and diabetic retinopathy, so a wider appreciation for the need for comprehensive eye care has also been lacking. There is a need for eye-care NGOs to engage with the health ministry from the outset, to ensure that their activities are aligned and integrated with health sector strategies and plans. If the government is involved, it is likely that eye care will be taken to scale. In my experience, governments are keen to see the impact of these interventions, for example whether they achieve high coverage, are cost–effective, increase access and improve health outcomes, before they are willing to invest resources in them.

Q: How does your work help to improve the quality of National Eye Health Programmes through evaluation?

A: There were common themes in almost all programme evaluations and reviews, such as those I did in Ethiopia, Kenya, South Africa, Uganda and the United Republic of Tanzania. We found that while programmes and projects had indicators to achieve, there was insufficient emphasis on quality indicators. There was no engagement by the programme implementers with the quality assurance section of the health ministries, which meant that the programme didn’t benefit from ongoing plans for quality improvement. In addition, while monitoring and evaluation frameworks tended to focus on clinical quality as measured by visual acuity, establishing quality indicators that defined a critical pathway of programming and intervention would have been useful. One of the key findings was that eye health was being delivered as a fragmented collection of activities. In most of these evaluations, I recommended that they establish an essential package of eye health services for primary and secondary levels of health care as part of minimum service delivery standards. This package would include: scope of service delivery, posts for human resources required, equipment, medicines, space, referral pathways and eye health information reporting.

Q: How would you assess overall progress in terms of the provision of eye care?

A: There has been an incremental growth in the scope and coverage of national eye health programmes in different regions. Five key challenges remain that need to be addressed in the sustainable development goals era. First: ensuring universal eye health coverage so that no one is left behind. Second: integrating eye health more formally in health sector strategies and plans. Third: improving eye health information processes as integral components of health information pathways. Fourth: improving sustainable development and deployment of human resources for eye health. Fifth: developing an inclusive eye health approach that addresses equity, gender, disability and vulnerable and excluded communities.

